# Higher amplitudes of visual networks are associated with trait- but not state-depression

**DOI:** 10.1017/S0033291724003167

**Published:** 2024-12

**Authors:** Wei Zhang, Rosie Dutt, Daphne Lew, Deanna M. Barch, Janine D. Bijsterbosch

**Affiliations:** 1 Department of Radiology, Washington University School of Medicine, St. Louis, MO, USA; 2 Biological Sciences Collegiate Division, University of Chicago, Chicago, IL, USA; 3 Center for Biostatistics and Data Science, Institute for Informatics, Data Science, and Biostatistics, Washington University School of Medicine, St. Louis, MO, USA; 4 Department of Psychiatry, Washington University School of Medicine, St. Louis, MO, USA; 5 Department of Psychological and Brain Sciences, Washington University in St. Louis, St. Louis, MO, USA

**Keywords:** brain network, depression, neuroimaging, PROFUMO, resting-state fMRI, state depression, trait depression, UK Biobank

## Abstract

Despite depression being a leading cause of global disability, neuroimaging studies have struggled to identify replicable neural correlates of depression or explain limited variance. This challenge may, in part, stem from the intertwined state (current symptoms; variable) and trait (general propensity; stable) experiences of depression.

Here, we sought to disentangle state from trait experiences of depression by leveraging a longitudinal cohort and stratifying individuals into four groups: those in remission (‘trait depression group’), those with large longitudinal severity changes in depression symptomatology (‘state depression group’), and their respective matched control groups (total analytic *n* = 1030). We hypothesized that spatial network organization would be linked to trait depression due to its temporal stability, whereas functional connectivity between networks would be more sensitive to state-dependent depression symptoms due to its capacity to fluctuate.

We identified 15 large-scale probabilistic functional networks from resting-state fMRI data and performed group comparisons on the amplitude, connectivity, and spatial overlap between these networks, using matched control participants as reference. Our findings revealed higher amplitude in visual networks for the trait depression group at the time of remission, in contrast to controls. This observation may suggest altered visual processing in individuals predisposed to developing depression over time. No significant group differences were observed in any other network measures for the trait-control comparison, nor in any measures for the state-control comparison. These results underscore the overlooked contribution of visual networks to the psychopathology of depression and provide evidence for distinct neural correlates between state and trait experiences of depression.

## Introduction

Depression is a global health challenge, emerging as the foremost cause of disability and affecting more than 300 million individuals worldwide (Friedrich, 2017). While there is widespread acknowledgment of depression as a disorder associated with dysfunctions of large-scale brain networks (Williams, 2016), existing research has been hindered by inconsistencies (Greene et al., 2022; Tozzi et al., 2020; Xia et al., 2019), lack of reproducibility (Kennis et al., 2020; Saberi, Mohammadi, Zarei, Eickhoff, & Tahmasian, 2022), and, at best, the ability to account for only a modest proportion of variance (Dutt et al., 2022; Schmaal, 2022; Winter et al., 2022). This prevailing uncertainty creates a substantial gap in our comprehension of the neural basis and potential etiology of this pervasive mental health condition. Addressing this knowledge deficit is important, as a deeper understanding of depression's etiological underpinnings can improve diagnostic, treatment, and prevention strategies.

At the functional neurocircuitry level, investigations using resting-state fMRI have revealed key insights into the differences between individuals with major depressive disorder (MDD) and healthy controls (Kaiser, Andrews-Hanna, Wager, & Pizzagalli, 2015; Mulders, van Eijndhoven, Schene, Beckmann, & Tendolkar, 2015). Importantly, previous review and meta-analysis studies on depression indicate hyperconnectivity within the default mode network (DMN) and hypoconnectivity within the central executive network (CEN) (Kaiser et al., 2015). However, recent meta- and mega-analyses present contradictory findings, reporting comparable connectivity patterns of the DMN and CEN between patients and controls (Javaheripour et al., 2021), or reduced connectivity in patients (Yan et al., 2019). Despite being less explored in previous neuroimaging studies on depression, disruptions in the functional connectivity of sensory and motor networks have also been implicated in MDD, albeit with similar inconsistency in directions of these connectivity disruptions both within sensory and motor networks or between these and other brain networks (Kang et al., 2018; Lu et al., 2020; Ray, Bezmaternykh, Mel'nikov, Friston, & Das, 2021; Wu, Lu, Kong, & Zhang, 2023; Wüthrich et al., 2023; Zeng et al., 2012; Zhu et al., 2021).

The varying findings across studies can be attributed, at least in part, to the diverse study designs employed. These include comparisons between patient and control groups (Flint et al., 2021; Winter et al., 2022), regressions against depression severity scores (Oathes, Patenaude, Schatzberg, & Etkin, 2015; Yoshida et al., 2017) or personality traits like neuroticism (Braund et al., 2022; Fournier et al., 2017; Steffens, Wang, Manning, & Pearlson, 2017), and investigations into heterogeneity (Dinga et al., 2019; Drysdale et al., 2017; Hannon et al., 2022; Wen et al., 2022; Yang et al., 2021). Moreover, the mixture of state depression (current symptom severity) and trait depression (long-term propensity for depression) further complicates study designs. While state and trait depression are often viewed as closely related and sometimes used interchangeably, biomarkers that differentiate between state and trait depression can serve distinct purposes in a clinical setting. For instance, trait biomarkers are instrumental in identifying individuals at risk, while state biomarkers help gauge treatment effectiveness and track patients' progress over time. This differentiation aligns with established categories of biomarkers outlined by the FDA-NIH Biomarker Working Group (2016). The failure to differentiate between state and trait depression likely contributes to inconsistencies in findings across the literature. Therefore, elucidating the distinct neural correlates of state *v.* trait depression is poised to not only enhance the utility of biomarkers but also offer clarity regarding the underlying neural mechanisms of the disorder.

To date, few studies have specifically examined state and/or trait depression. These investigations often involve comparisons between participants in remission (trait) and those experiencing a current episode of depression (state) (Admon et al., 2015; Ming et al., 2017), or employ longitudinal designs to assess changes pre- and post- pharmacological interventions (state) (Delaveau et al., 2011). Unfortunately, however, these studies have yielded mixed results. In one study, the ventromedial prefrontal cortex and precuneus were suggested to signify trait markers of depression (Ming et al., 2017), while another study indicated altered activity in the same regions following antidepressant drug treatment, implying state-dependent changes (Delaveau et al., 2011). Besides the heterogeneity in analytical approaches that is well known to hinder reproducibility and replicability in functional MRI studies (Adali & Calhoun, 2022; Botvinik-Nezer et al., 2020), insufficient power might also have contributed to the inconsistency in these findings, as larger sample sizes are required to detect small but meaningful effects in brain-behavior associations (Marek et al., 2022; Ooi et al., 2024).

Insights into trait-like depression experiences may also be gained from investigations focusing on individuals with a familial history of, or genetic predisposition to, mood disorders. A recent review reported aberrant connectivity between the amygdala and a wide range of brain regions/networks in infants (Posner et al., 2016; Qiu et al., 2015), 5-year old children (Soe et al., 2017), and adolescents (Fischer, Camacho, Ho, Whitfield-Gabrieli, & Gotlib, 2018; Singh, Leslie, Packer, Weisman, & Gotlib, 2018), as well as altered DMN connectivity in adolescents (Bellgowan et al., 2015; Chai et al., 2016) with elevated risks of depression (Nazarova, Schmidt, Cookey, & Uher, 2022). Another study linking polygenic risk scores for depression and resting-state connectivity in young adults further highlighted alterations in subgenual anterior cingulate cortex (sgACC)-based networks as depression markers (Chen et al., 2024). However, the significant heterogeneity between these studies (e.g. variability in preprocessing and analytical methods) and the limited number of replicated findings make it challenging to conclude the specific regions affected within these resting-state networks (Nazarova et al., 2022).

Collectively, the inconsistency in prior findings suggests that the potentially dissociable neural correlates of state *v.* trait depression remain inadequately understood.

Leveraging the UK Biobank (UKB) data, the present study aimed to identify potentially dissociable resting-state functional correlates of state and trait depression. Inspired by previous work (Admon et al., 2015; Delaveau et al., 2011; Ming et al., 2017), we employed longitudinal data of depression severity to differentiate state and trait experiences. This approach resulted in indications of a high-level propensity to depression during remission (trait) and substantial fluctuations in symptom severities between two time points (state), respectively, effectively dissociating state from trait depression. We further applied a state-of-the-art data-driven decomposition method to identify large-scale brain networks from the resting-state fMRI data and estimated the amplitude (i.e. network strength), connectivity (i.e. between-network temporal correlations also known as network matrices), and spatial overlap (i.e. shared regions between network spatial maps) of these brain networks. Given the distinct temporality in state- *v.* trait-depression experiences (i.e. changing *v.* stable) and drawing from literature that suggests dynamic alterations in functional connectivity alongside more enduring spatial configurations of brain networks (Harrison et al., 2020), we hypothesized that longitudinal changes in functional connectivity of large-scale brain networks would be associated with changes in depression symptom severity (i.e. state depression), whereas the spatial organizations of those networks would be related to trait depression experiences.

## Methods

The group sampling and statistical analysis plans described below were pre-registered at the Open Science Framework (Zhang & Bijsterbosch, 2023).

### Participants

**Figure 1.**
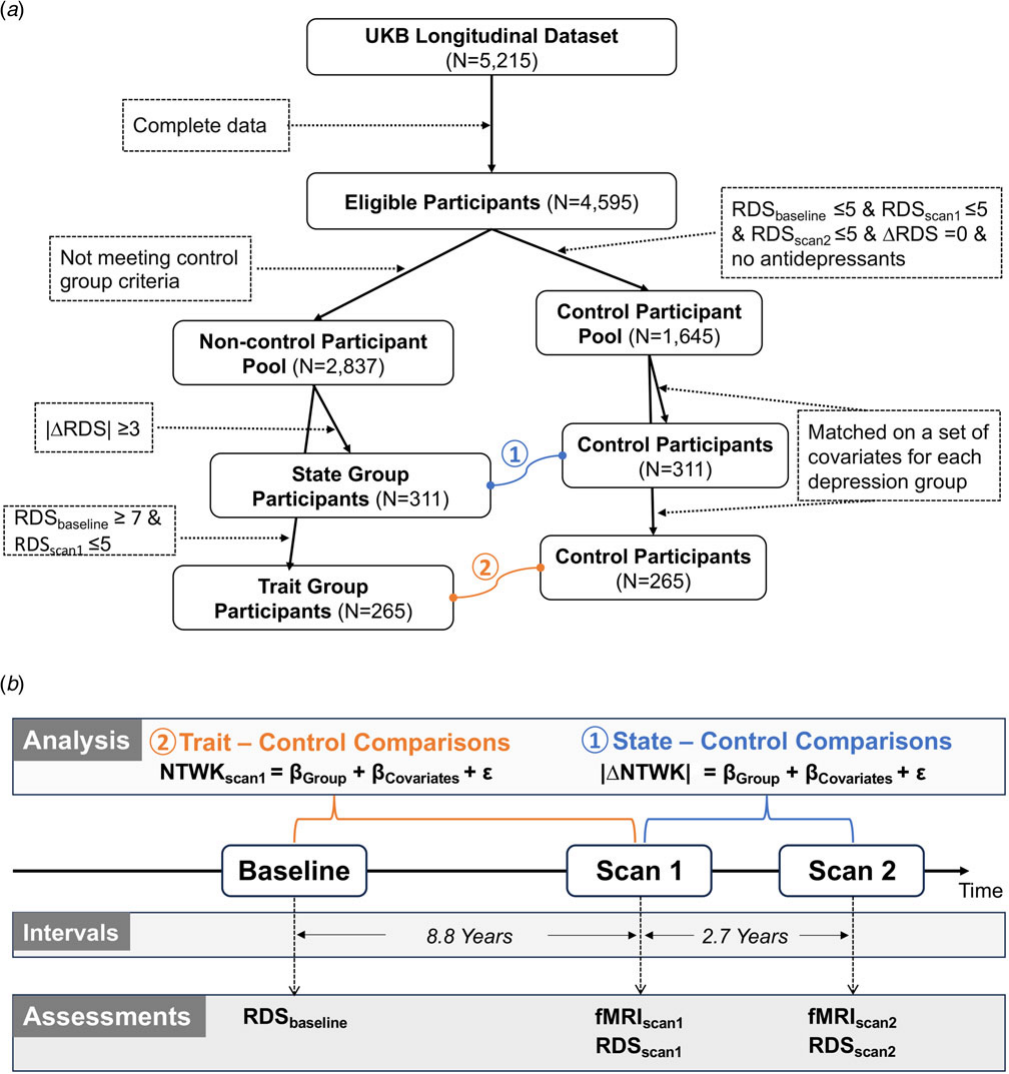
Flowchart of the study sample (*a*) and schema of analysis pipeline (*b*). Specifically, the RDS_baseline_, RDS_scan1_, and RDS_scan2_ represent sum scores of recent depressive symptoms (RDS) obtained at different time points, with subscripts indicating the assessment time, whereas |ΔRDS| denotes the absolute longitudinal change score of RDS between two neuroimaging scans. The final sample consisted of two pairs of matched groups, connected by curved lines. Group comparisons between the matched state-control (①) and trait-control (②) were performed separately, utilizing brain network measures (NTWK) assessed at different time points. Importantly, in trait-control comparisons, the network measures at scan1 (NTWK_scan1_) were considered dependent variables, while in state-control comparisons, the absolute values of longitudinal changes in the network measures (|ΔNTWK|), were included as dependent variables. All these dependent variables were modeled as a function of the group variable (e.g. state v. control), while accounting for covariates.

Out of *N* = 5215 longitudinal UKB participants, *N* = 4595 had complete longitudinal neuroimaging and depression data. To discern state and trait depression, we created two corresponding groups and matched controls. The state depression group (*N* = 311) was based on change scores in depression severity between two assessments (i.e. scan 1 and scan 2), while the trait depression group (*N* = 265) had high baseline (i.e. high propensity) but low current symptom severity (i.e. remission at scan 1). Their respectively matched controls (*N* = 311 and *N* = 265) had consistently minimal severity scores across all time points (see detailed group definitions in *section 2*). The resulting sample (*N* = 1030) included *n* = 33 overlaps between the state and trait groups and *n* = 89 overlaps between the two matched control groups (see study sample flowchart in Fig. 1a). As we performed statistical analyses separately for state-control and trait-control comparisons, these overlapping participants were included in each group's respective comparison analysis. Demographics are summarized in Table 1.

**Table 1.** Demographics of matched depression and control groups

	Matched state-control groups			Matched trait-control groups		
	State (*n* = 311)	Control (*n* = 311)	Stats^a^	Trait (*n* = 265)	Control (*n* = 265)	Stats^a^
*N*_Female_ (%)	196 (63)	217 (70)	3.18	167 (63)	179 (68)	1.20
Mean_age-baseline_ (s.d.)	51.21 (6.79)	51.38 (7.29)	0.32	52.19 (6.49)	52.06 (7.26)	−0.23
Mean_age-scan 1_ (s.d.)	59.95 (6.58)	60.22 (7.26)	0.49	61.01 (6.55)	60.81 (7.26)	−0.36
Mean_age-scan 2_ (s.d.)	62.61 (6.39)	62.83 (4.77)	0.41	63.43 (4.40)	63.43 (7.04)	−0.53
Mean_RDS-baseline_ (s.d.)	6.39 (2.48)	4.77 (0.42)	−12.38**	7.82 (1.33)	4.40 (0.49)	−39.00**
Mean_RDS-scan 1_ (s.d.)	7.14 (2.73)	4.31 (0.46)	−18.09**	4.57 (0.50)	4.56 (0.50)	−0.71
Mean_RDS-scan 2_ (s.d.)	7.30 (2.91)	4.31 (0.46)	−17.97**	5.26 (1.48)	4.56 (0.5)	−7.79**
Mean_interval_ (s.d.)^b^	949 (360)	973 (423)	1.09	3221 (492)	3214 (514)	−1.15
*N*_Antidepressants_^c^	79	0	NA	38	0	NA

*N*, sample size; s.d., standard deviation; RDS, Recent Depressive Symptom scale; NA, not applicable.

a χ^2^ test was conducted to examine between-group difference in sex, and unpaired *t* tests to examine numerical variables.

b Intervals (in days) between baseline assessment and scan 1 for state, between scan 1 and scan 2 for trait.

c Number of participants on antidepressants in each group (see online Supplemental Materials for the full list of medications). By definition, no control participants had antidepressants.

** *p* < 0.0001.

### Defining Depression and Control Groups

#### State depression group

Longitudinal data were used to capture changes in state depression, identifying participants with a large (⩾3 points) absolute change in longitudinal score of recent depressive symptoms (RDS) between the first and follow-up imaging assessments (i.e. scan1 and scan2). The absolute change (|ΔRDS|) was chosen to allow for symptom severity changes in both increased and decreased directions, capturing the full spectrum of state-dependent fluctuations. The RDS is a 4-item scale with scores ranging from 4 to 16, validated against standardized scales of depression (Dutt et al., 2022). The 3-point change threshold, representing 25% of the full 12-point scale for RDS, signifies clinically meaningful changes in state depression while maximizing sample size (see online Supplemental Materials Table S1). As a reference, a 25% change on the PHQ scale (0–27) would exceed the 5-point cut-off for mild depressive symptoms (Kroenke, Spitzer, & Williams, 2001). *N* = 311 participants met the |ΔRDS| >3 inclusion requirement for the state depression group.

#### Trait depression group

Trait depression, reflecting a long-term tendency to experiencing depressive symptoms (Klein, Kotov, & Bufferd, 2011) was operationalized in our study as individuals with prior depression but in remission at the time of scanning (i.e. scan 1). Specifically, participants with an RDS score at the baseline equal to or above 7 (RDS_baseline_ ⩾ 7) AND an RDS score at the first imaging assessment equal to or below 5 (RDS_scan1_ ⩽ 5) were included in the trait depression group. Notably, our previous work has mapped RDS onto PHQ-9 (Dutt et al., 2022), where an RDS score of seven corresponds to PHQ = 5, the clinical threshold for mild depression (Kroenke et al., 2001). Additionally, an RDS score of five represents a minimal degree of depression and RDS_scan1_ ⩽ 5 indicates a remission status at the first imaging assessment. *N* = 265 participants met the RDS_baseline_ ⩾ 7 & RDS_scan1_ ⩽ 5 inclusion requirements for the trait depression group.

Based on these grouping criteria, we included different groups of participants for trait *v.* state depression groups. This deliberate separation was chosen to empirically dissociate state from trait experiences of depression, allowing for clearer interpretations of results.

#### Control participants and group matching

A pool of potential control participants was identified, meeting criteria of minimal depression scores across all three time points (RDS_baseline_ ⩽ 5 & RDS_scan1_ ⩽ 5 & RDS_scan2_ ⩽ 5), and no change in depression score between scan 1 and scan 2 (ΔRDS = 0). These individuals were not on antidepressant medication at any time point either, ensuring a healthy reference group (see list for antidepressants in online Supplemental Materials). We identified a total of *N* = 1448 participants meeting these criteria. From this total pool, we separately selected the equivalent number of participants for both the state and trait depression groups. These selections were made to ensure matching for each depression group on sex, age, in-scanner head motion (i.e. averaged relative framewise displacement), scanning site, and alcohol intake frequency at the first imaging assessment (i.e. at scan 1), thus minimizing potential confounding effects on between-group differences under investigation.

Matches were identified using propensity scores with an optimal matching algorithm, selected from several algorithms (see matching diagnostics in online Supplemental Materials Table S3). The matched groups demonstrated improved covariate balance for all variables of interest, with no significant group difference observed between the depression groups and their respective matched controls (all *p*'s > 0.05; online Supplemental Materials Table S4).

### fMRI data acquisition and preprocessing

The UKB acquired resting-state neuroimaging data using a 3 T Siemens Skyra (2.4 mm isotropic voxel size, TR = 0.735 s, multiband factor 8). The detailed scanning protocols are documented online (https://biobank.ctsu.ox.ac.uk/crystal/refer.cgi?id=2367). Our study used preprocessed data that were released via the UKB showcase. The preprocessing steps included distortion correction, motion correction, high pass temporal filtering, and BOLD signal denoising using ICA-FIX (details in (Alfaro-Almagro et al., 2018).

#### Brain networks identified by probabilistic functional modes estimation

Probabilistic functional modes (PROFUMO) is a hierarchical Bayesian approach to decompose resting state neuroimaging data into a set of modes, representing resting-state brain networks (Harrison et al., 2015, 2020). Each mode or network is described by a spatial map, its associated time course, and signal amplitude. At the subject level, a network matrix (i.e. connectivity matrix) is estimated as the partial correlation between pairwise mode time courses, capturing between-network similarities in their temporal dynamics. These PROFUMO outputs encompass both the temporal and spatial characteristics of each mode or network: the spatial map demonstrates the anatomical configuration of each probabilistic network, the amplitude reflects the overall signal fluctuation for each network, and the network matrix represents the overall connectivity patterns among all modes or networks per subject. Notably, each of these PROFUMO outputs is estimated separately per longitudinal scan and simultaneously at the group and individual levels. PROFUMO offers advantages in effectively capturing spatial overlaps in network structures that indicate shared spatial organization across networks (Bijsterbosch, Beckmann, Woolrich, Smith, & Harrison, 2019), and demonstrates increased sensitivity in discerning individual-specific network configuration (Bijsterbosch et al., 2018; Harrison et al., 2020). In practice, spatial overlaps between each pair of PROFUMO modes or networks are estimated by taking the subject-specific 3-dimensional spatial map for each mode, vectorizing the 3-dimensional spatial matrix of each mode, and then calculating the Pearson's correlation between the vectors for each pair of modes. To maintain consistency across state and trait analyses, we merged resting-state fMRI data from all four groups, resulting in a sample size of 1030 unique individuals. Each participant contributed data from two time points (scan1 and scan2), totaling 2060 scans for the final PROFUMO decomposition.

In the main analysis, we set the PROFUMO mode dimension to 15, aligning with our focus on large-scale networks. After removing two spurious modes (online Supplemental Materials), we included 13 meaningful brain networks for statistical analyses and used each of the three PROFUMO outputs per network as dependent variables. To ensure robustness, we also examined 10 and 20 dimensions, validating significant findings from the 15-dimensional outputs. This adjustment from our preregistration, originally focused on 20-dimensional networks, aimed to streamline multiple-testing comparisons, and emphasize a low-dimensional decomposition into large-scale canonical resting-state networks.

### Statistical analysis

Using linear regression models, separate analyses compared group differences in neural correlates between each of the two depression groups and their respective control groups. Specifically, state-control comparisons assessed longitudinal changes in network measures between two neuroimaging scans, while trait-control comparisons considered network measures at scan1 as the dependent variables (see Fig. 1b). These dependent variables comprised PROFUMO outputs, including mode amplitude (indicating overall signal fluctuation), network matrix (reflecting partial correlation between mode timeseries), and spatial overlap matrix (illustrating full correlation between mode spatial maps).

#### Main analyses

The primary analysis for state depression involved computing longitudinal changes in each of the three brain network measures (i.e. PROFUMO outputs) between two scans (i.e. scan 2 minus scan 1). The absolute values of these changes served as dependent variables in subsequent group-comparison analyses. In the main analysis for trait depression, the network measures at scan 1 were utilized as dependent variables in the group-comparison analyses. Thus, separate regression analyses were conducted for state-control and trait-control group comparisons, utilizing different dependent variables (see analysis equations in Fig. 1b). Within each comparison, we performed statistical analysis separately for each class of PROFUMO outputs. Covariates, including sex, age, in-scanner head motion, scanning site, alcohol intake frequency, time interval between two scans (only for state-control comparisons), and use of antidepressants, were adjusted in all analyses.

To address multiple testing concerns within each class of three PROFUMO outputs, false discovery rate (FDR) correction was applied. This approach was chosen to accommodate distinct hypotheses for different PROFUMO outputs.

The number of separate univariate analyses was determined by the PROFUMO dimensionality. In our main analyses (using 13 meaningful modes), we obtained 13 mode amplitudes, 78 temporal edges from connectivity matrix, and 78 spatial edges indicative of spatial overlaps between modes from spatial correlation matrix. These edges represent unique pairwise correlation coefficients derived from the upper or low triangle of the connectivity or spatial correlation matrices (i.e. 13 × 12/2 = 78), constructed from the temporal or spatial correlations between each pair of timeseries or spatial maps of modes.

#### Follow-up analyses

Several follow-up analyses were conducted to further validate the statistical significance of the group comparison results from our main analyses.

First, we performed regression analyses to investigate symptom magnitude-dependent effects within depression groups in case significant group differences were observed in any of the three PROFUMO outputs. This involved separate analyses for each significant result from the group comparisons among individuals within the relevant depression group. For example, if individuals with trait depression exhibited larger connectivity strength than their control counterparts, the subsequent tests would examine whether, among individuals with trait depression, greater connectivity strength was associated with higher symptom severity.

Secondly, we conducted follow-up analyses aiming to identify robust findings across different dimensionalities. We first identified the ‘best-matched’ mode(s) from 10- and 20-dimension decompositions for the target modes from the 15-dimension decomposition, using Cosine similarity coefficients (see details in online Supplemental Materials). Subsequently, we repeated group comparisons on the identified modes from 10 and 20 dimensionalities to confirm the persistence of significant results observed in the 15-dimensionality. While our pre-registration suggested the Hungarian algorithm (Kuhn, 1955) for mode matching, we adjusted our strategy to prioritize replicating findings for specific modes, favoring spatial similarity over the complete data matching. This choice prevents issues with imbalanced dimensionalities (e.g. 10 *v.* 15), ensuring effective mode matching.

Thirdly, the state-depression group was defined using a binary approach (i.e. above a certain symptom threshold), which does not capture associations on a continuous scale. Additionally, our statistical analysis initially considered only the absolute values of brain measures, potentially obscuring directional insights. To address these limitations, we repeated the statistical analysis for the state-depression group using the original change score (i.e. without taking the absolute value). Given the binary definition, symptom change scores for the state-depression group likely follow a binomial distribution. Therefore, we repeated the analyses on two additional samples: the entire study cohort (*N* = 1030) and the combined state-depression and trait-depression groups (*N* = 576).

## Results

### Characteristics of participants in each group

With our operationalized definitions of the state and trait depression groups, participants in the state group showed higher RDS scores across all time points, whereas participants in the trait group only reported higher RDS scores compared to controls at baseline and scan 2 (*p*'s < 0.0001), with comparable RDS scores to controls at scan 1, indicating the intended remission status (Table 1).

Importantly, these operational definitions achieved the desired state-trait dissociation by reducing the known high correlations between these two constructs. Specifically, for the participants in the state group, the longitudinal changes in RDS between two scans (ΔRDS), reflecting state-dependent depression experiences, showed a negligible correlation (*r* = −0.01) with the baseline RDS (RDS_baseline_). This substantially reduced the initial correlation between the ΔRDS and RDS_baseline_ (*r* = 0.291) in the full UKB sample (*n* = 4595). A similar state-trait dissociation was observed for the participants in the trait depression group: The RDS_baseline_, used to define trait experiences, exhibited only a correlation of *r* = 0.03 with the RDS at scan1 (RDS_scan1_), compared to the initial *r* = 0.52 in the full UKB sample. The RDS_scan1_ here indexed the present depression symptoms of the participants from the trait depression group at the time of scan 1, when the brain network measures were assessed for these participants.

Furthermore, the control participants exhibited similar time intervals between different assessments, along with demographic and several other variables that were matched between the depression and control groups (Table 1; online Supplemental Materials Table S4).

### Decomposed PROFUMO modes

At the group level, a total of 15 probabilistic modes were estimated across all depression and control participants using both scan 1 and scan 2 data, 13 of which were identified as large-scale brain networks representing meaningful brain signals (see full decomposition in online Supplemental Materials Fig. S1). The spatial distributions of these probabilistic modes highly resembled canonical brain networks including the default mode, frontoparietal, visual and motor networks. Similar resemblances were also observed in 10- and 20-dimension decompositions, except that some of the networks in one decomposition appeared to merge into one or split into two or more modes in another decomposition (online Supplemental Materials Figs S2 and S3).

### Group comparisons in neural correlates

**Figure 2.**
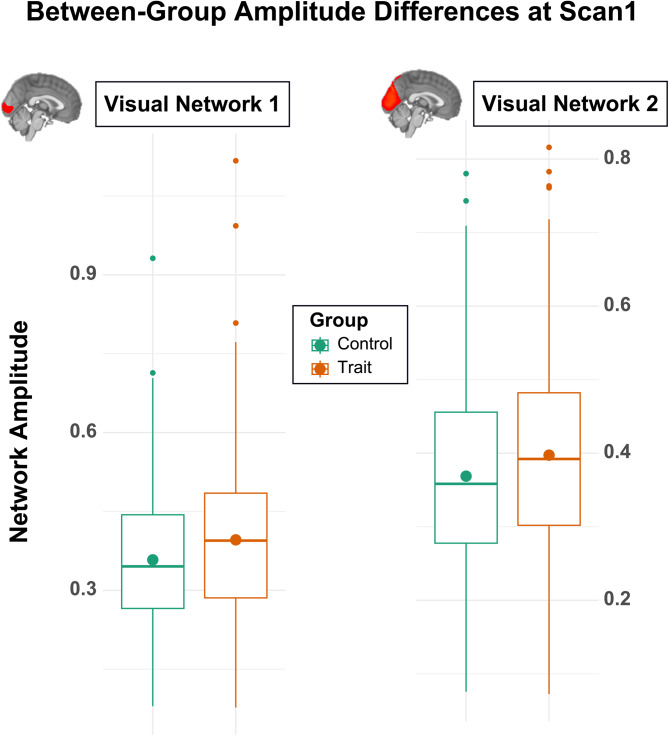
Box plots showing group differences in amplitude of two visual networks at the time of scan 1, between individuals with trait experience of depression (in orange) and control participants (in green), with higher mean amplitudes in both networks, as annotated by filled circles, in the trait depression group. Note, separate *Y*-axis scales were used to highlight the group mean differences within each comparison.

Individuals experiencing trait depression exhibited significantly higher amplitude in two visual networks in contrast to the matched control participants (Fig. 2). Anatomically, visual network 1 predominantly involves the inferior division of the lateral occipital lobe, including the occipital pole, and extends ventrally into the lingual gyrus. For visual network 2, the strongest signal was observed in the superior division of the lateral occipital lobe. This network further extends ventrally across the inferior division of the lateral occipital lobe, lingual gyrus, and fusiform gyrus, while also extending dorsally into the superior parietal lobe. These observed effects of increased amplitude were robust against the inclusion of all covariates including antidepressant usage (*β*_visual1_ = 0.045, *β*_visual2_ = 0.034, FDR corrected *p*'s < 0.05). These findings also replicated in the follow-up analyses using PROFUMO outputs from the 10- and 20-dimension decompositions (*β*'s > 0.02, *p*'s < 0.03; online Supplemental Results Table S5). However, the magnitude of the mode amplitude within the visual networks was not associated with depression symptom severity at baseline among individuals within the trait group (*β* = −0.009, *p* > 0.8). Further, we did not find significant group differences in spatial overlaps or network matrices for the trait analyses (all FDR corrected *p*'s > 0.05).

After corrections for multiple testing and potential confounding effects, we did not find significant group differences in any absolute longitudinal changes in PROFUMO outputs (amplitude, network matrix, and spatial overlap) between individuals from the state-depression group and the matched controls (all FDR corrected *p*'s > 0.05).

In our follow-up analyses, after applying FDR corrections, no significant associations were observed between symptom change scores and any of the PROFUMO outputs. This null finding held true for the state-depression group alone, the combined trait- and state-depression groups, and the entire study cohort (all FDR corrected *p*'s > 0.05).

## Discussion

In the current study, we designed group contrast that effectively disassociated the neural correlates of state and trait experiences of depression. Using matched control participants as the reference, our results showed that greater amplitudes in two visual networks were associated with trait depression and that these associative effects were robust against relevant confounders and across different dimensionalities of brain networks. These findings demonstrate that the overall BOLD signal fluctuations within the visual networks may serve as a potential biomarker for trait depression.

### Visual cortex and trait depression

Depression is a mental disorder characterized by dysfunctional brain networks (Williams, 2016). Although the majority of the major depressive disorder (MDD) literature focuses on alterations in the default mode, salience, and central executive networks (Kaiser et al., 2015), there is increasing evidence linking depression to structural and functional alterations in sensory and motor networks, including visual networks (Wu et al., 2023; Zhukovsky et al., 2021).

Structurally, individuals with MDD often exhibit increased volume (Ancelin et al., 2019), greater asymmetry (Maller et al., 2014), and altered surface area (Schmaal et al., 2017) in the occipital cortex compared to healthy controls. These structural alterations have been further associated with symptom recurrence and duration in MDD patients (Kang et al., 2023). Similar structural alterations have also been reported for individuals with potential trait depression (Nazarova et al., 2022). For instance, healthy adolescents with high familial risks for depression showed reduced cortical thickness in the lateral occipital gyrus (Foland-Ross, Behzadian, LeMoult, & Gotlib, 2016), and lower white matter integrity in inferior fronto-occipital fasciculi (Huang, Fan, Williamson, & Rao, 2011). Furthermore, individuals at high familial risk for developing MDD demonstrated expanded cortical thinning in a wide range of brain regions, including the inferior occipital gyrus, which was also correlated with current symptom severity (Peterson et al., 2009).

At the functional level, MDD patients demonstrate various abnormalities within the visual network. This includes decreased regional homogeneity (Peng et al., 2011; Yan et al., 2021), lower nodal efficiency in occipital areas (Xu et al., 2022), aberrant resting-state functional connectivity within the network (Lu et al., 2020; Yu et al., 2019), and disruptions in connections between the visual network and other brain networks (Desseilles et al., 2011; Kaiser et al., 2015; Le, Borghi, Kujawa, Klein, & Leung, 2017; Yan et al., 2019; Young et al., 2023). Moreover, depressed individuals exhibit abnormal filtering of irrelevant information in the visual cortex (Desseilles et al., 2009) and altered activity patterns in visual association areas affecting working memory (Le et al., 2017).

Although fewer studies have examined brain network activity and connectivity in at-risk individuals, current evidence suggests that familial risks for depression may heighten emotion-related visual processing, with increased activation within the visual cortex and its connectivity with other brain regions, such as the amygdala (Wackerhagen et al., 2017). Additionally, connectivity between the visual network and prefrontal regions has been shown to predict the onset of internalizing disorder (i.e. depression and anxiety) for individuals with a parental history of these conditions (Pawlak, Bray, & Kopala-Sibley, 2022). In contrast to adolescents with low familial risk for depression, those at high risk have demonstrated a stronger association between follow-up symptom severity and the baseline connectivity of sensory/somatosensory networks with amygdala/striatal regions (Holt-Gosselin et al., 2024). Abnormal activation patterns in the visual cortex have also been observed in remitted depression patients during emotion regulation (van Kleef et al., 2022) and rumination (Burkhouse et al., 2017). Furthermore, increased amplitude of low-frequency fluctuation (ALFF) in the occipital cortex has been linked to patients with remitted depression in contrast to healthy controls (Cheng et al., 2019).

These findings collectively underscore the crucial role of the visual cortex in the psychopathology of depression (Friberg & Borrero, 2000; Salmela et al., 2021). Such alterations may be attributed to reduced concentrations of the neurotransmitter GABA in MDD patients, leading to deficits in the inhibition or suppression of relevant visual information processing (Price et al., 2009; Song et al., 2021). Since brain network amplitudes primarily reflect the level of synchronous activation among functionally connected regions (within a network), and are closely related to functional connectivity between networks (Lee et al., 2023), our observation of increased amplitude in visual networks indicates heightened synchrony among regions within these networks, and potentially aberrant connectivity between visual networks with other brain regions and/or networks. The anatomical location of these two visual networks (e.g. lateral occipital, lingual, and fusiform gyri) also aligns well with prior findings highlighting the involvement of these regions in abnormal visual information processing in depression (Chen et al., 2019; Wu et al., 2023), which may contribute to altered visual perception and associated cognitive and emotional impairments (Atchley et al., 2012; De Zorzi et al., 2020; Fam, Rush, Haaland, Barbier, & Luu, 2013; Golomb et al., 2009; Salmela et al., 2021; Valt et al., 2022).

Yet, our study found no association between the magnitude of amplitude increase and symptom severity among individuals with trait depression, likely due to limited variability in symptom severity within this group. Nearly half of the individuals (120 out of 265) in this depression group exhibited mild-level symptoms (RDS = 7), which was the minimum criterion for inclusion in the trait group. However, despite the limited variability, the propensity for depression was sufficient at the group level to be associated with differences in brain function, evident in the between-group differences in visual network amplitude.

### Null findings for state depression

In contrast to our hypothesis, no significant differences in network connectivity or other measures were found between the state depression group and the control group. These null findings may have resulted from several factors.

Firstly, the state experience in our study was defined as substantial fluctuations in symptom severity between two imaging scans. While the overall symptom severity score provides insight into the general experiences of state depression, it may not capture fluctuations in specific symptoms. This limitation makes the investigation into associated brain measures susceptible to individual heterogeneity, as network measures for different symptoms at the individual level may not converge at the group level.

Secondly, we opted for a cut-off score of 3 to define the state experience, aiming at capturing meaningful changes in symptoms, equivalent to 25% of the full 12-point scale. This choice was made to also maximize the sample size with sufficient statistical power to ensure robustness in detecting effects of interest. For example, increasing the cut-off score to 4, would have reduced the combined sample size to 256 (i.e. *n* = 311 to *n* = 128 for trait depression and control groups each; online Supplemental Materials Table S1), potentially leading to underpowered analyses. Retrospective power analysis using the reduced sample size showed 70% power to detect a small group effect at *β* = 0.05, slightly higher than *β*_visual1_ = 0.045 in our main findings, compared to the 93% power obtained from the current combined *n* = 622. However, it is possible that this mild cut-off may have been insufficient to detect state-related alterations in network connectivity or other brain measures.

Additionally, our current definition of state depression considered the magnitude of symptom fluctuations, combining both positive and negative changes in symptom severity between two imaging scans. If different brain networks are engaged in different directions of state changes, our approach might fail to detect group-level effects due to a potential mixture of results. Unfortunately, our sample size for the state depression participants was nearly halved when considering two separate groups with opposing change directions (*n* = 154 for negative changes and *n* = 157 for positive changes), leaving separate testing underpowered. Although we partially addressed the sample size issue by combining the state- and trait-depression groups (*N* = 576) or using the entire study cohort (*N* = 1030) in our follow-up tests, no significant associations were found between symptom changes and any PROFUMO brain measure changes after accounting for multiple testing and confounding effects.

Lastly, given the dynamic nature of state depression experiences, the use of evoked study designs might offer greater sensitivity in capturing alterations in state-related neural correlates. Recent meta-analysis studies seem to support this notion, revealing reduced brain activation in a wide range of cortical and subcortical regions for MDD patients during emotional processing after antidepressant treatment in contrast to the baseline (Delaveau et al., 2011). Additionally, altered activity patterns have been observed in tasks related to emotional processing or executive functioning tasks, or across aggregated tasks from these two domains (Gray, Müller, Eickhoff, & Fox, 2020).

### Null findings in the default mode network (DMN)

Our current investigation did not find DMN differences in any PROFUMO output for either the trait or state comparison. This seemingly surprising result contrasts with the prior implications of this brain network in relation to depression. It is important to note, however, that previous research has also yielded inconsistent and/or contradictory findings regarding the role of the DMN in MDD (Javaheripour et al., 2021; Kaiser et al., 2015; Mulders et al., 2015; Yan et al., 2019). This inconsistency underscores the challenges in identifying robust neural correlates of depression across studies.

Despite employing a data-driven approach to identify brain networks including the DMN (i.e. mode 5 in online Supplementary Fig. S1), and utilizing data from a large-scale prospective epidemiological resource (Sudlow et al., 2015) to dissociate neural correlates of state and trait depression, our null findings align with the broader inconsistencies observed in the literature regarding alterations in the DMN and other brain networks in previous studies.

### Limitations

The current study has several limitations. First, our study sample is a subset from the UK Biobank study, comprising specific age groups (i.e. middle to older adults) and predominantly White (Sudlow et al., 2015). This limits the generalizability of our findings to more diverse populations with different demographic characteristics. Second, our groups for state and trait depression were defined using the overall symptom severity, which lacks the precision to identify brain correlates of individual symptoms known to be heterogeneous. Future research should consider addressing the variability in clinical presentations of depression to potentially enhance the reproducibility of brain association findings (Greene et al., 2022; Hannon et al., 2024; Kennis et al., 2020; Winter et al., 2022). Additionally, while we carefully generated two control groups and matched them for each of the target depression groups based on a set of crucial confounding variables, our analyses for group comparisons did not include psychosocial or lifestyle factors that can contribute to individual variations in depression symptoms (Aguilar-Latorre, Algorta, Navarro-Guzmán, Serrano-Ripoll, & Oliván-Blázquez, 2022; Remes, Mendes, & Templeton, 2021; Sarris, O'Neil, Coulson, Schweitzer, & Berk, 2014; Zhao et al., 2023). These additional factors may also influence the associative effects under investigation, and future studies should consider integrating them into analysis. Lastly, although we employed a data-driven approach to identify large-scale brain networks, this method appeared to favor the discovery of networks involving cortical regions over subcortical ones. These networks showed higher signal intensities predominantly in cortical areas, likely due to higher signal-to-noise ratios. Given the documented structural (Ho et al., 2020; Schmaal et al., 2016) and functional alterations (Gray et al., 2020; Miller, Hamilton, Sacchet, & Gotlib, 2015; Xiong et al., 2021) in subcortical regions in MDD, it may be prudent to explore supplementary approaches to better detect brain networks involving subcortical regions in the future.

## Conclusion

Incorporating pre-registered hypotheses and methods, this study aimed to examine the potentially distinct neural correlates of state and trait experiences of depression. We observed significantly higher amplitude in two visual networks for individuals in the trait depression group compared to controls. No significant differences in network measures were found in relation to state depression. Our findings suggest potentially altered visual information processing for individuals with a persistent tendency to experience depressive symptoms. These results highlight the potential contribution of visual networks to the psychopathology of depression that has been largely overlooked in the literature and provide evidence for neural correlates specific to trait experiences of depression.

## Supporting information

Zhang et al. supplementary material Zhang et al. supplementary material

## Supplementary material

The supplementary material for this article can be found at https://doi.org/10.1017/S0033291724003167
